# The Uses of the Chloride of Sodium

**Published:** 1851-09

**Authors:** W. B. Herrick

**Affiliations:** Prof. of Anatomy and Physiology in Rush Medical College, and one of the Surgeons to the Institution


ARTICLE VI

*The Uses of the Chloride of Sodium, with Notes of Cases Treated in the Illinois General Hospital.* By W. B. HERRICK, M. D., Prof. of Anatomy and Physiology in Rush Medical College, and one of the Surgeons to the Institution. Reported by H. A. Johnson, A. B. and Interne.

During the spring and summer, about ten cases of well marked Intermittent fever have been treated in the Hospital by the use of Chloride of Sodium. There have also been several cases of Typhoid Fever treated by the same article. So far as it has been tried, it seems to possess valuable medicinal properties, and if future experiments should confirm the results already obtained both here and elsewhere, we may expect that in intermittents the use of Quinine will soon be superseded by this simple and cheap remedy. The observations already made are not, perhaps, sufficiently numerous to warrant a definite conclusion in regard to this agent, but in order that others may be induced to try it, the two following cases are presented.

*Case First.*—R. S., an English laborer, aged 21, was admitted to the Hospital June 18th. About two months since he began to feel unwell, had head-ache, pain in the back, giddiness upon rising suddenly, with chill and fever; during the last week has had an ague fit every day.

Upon examination, the tongue was found covered with a light, yellowish fur; the bowels somewhat constipated; but very little appetite; pulse small and weak; skin cold, moist and dark; conjunctiva yellow; the spleen much enlarged, with tenderness upon pressure over the region of that organ; the general appearance of the patient indicated debility.

Prof. Herrick remarked, that this case presents us with a complication very often seen. The enlargement of the spleen is the result of the congestion of that organ, repeated with the occurrence of each chill. The first indication then, is, to arrest the exacerbations. This may be done either by making an impression on the nervous system, according to the general explanation, with Quinine and Opium, stimulating it to overcome the habit into which it seems to have fallen, and thus restoring an equilibrium to the circulation, or by attending immediately to the vascular system, improving the quality of the blood, so that it may be more efficient in removing from the system effete and morbid matter, the presence of which, it is believed, gives rise to the disease before us. In accomplishing this, we shall succeed best by introducing substances to prevent the further destruction of the globules of the blood, and at the same time furnishing the materials for the manufacture of a fresh supply of this constituent. If this can be effected, we may confidently expect, not only that the priodicity of the disease will be broken up, but that, the cause being removed, the disease itself must yield; and the system, instead of being depressed by the chemical or physical forces, must assume its wonted vigor, in obedience to the vital force, and in accordance with the laws of life. Chloride of Sodium is known to possess the property of preserving the blood globules; it is an alterative and tonic, and is also claimed to possess a specific influence in arresting the exacerbations of Intermittents. We have used it in several cases, and always with the most complete success. ℞ Chloride of Sodium ʒjj, in mucilage of Gum Arabic twice daily.

June 19. Had no chill since taking the Chloride of Sodium, feels much better, no head-ache—continue treatment.

June 22. Had a slight chill Friday, the 20th, but has felt well since; appetite begins to improve, tongue clean, tenderness over the region of the spleen much diminished, and some reduction in the size of that organ. Dr. H. remarked, that the disease might be considered as broken up. The only remaining indication, is to build up the system by the use of tonics and agents that favor the introduction of nutritious materials.

℞ Chlo. Sodii gr. X.
Ferri. Carb. grs. V.
M. Sum. three time daily before eating.

℞ Acidum Nitricum fʒi.

Acidum Muriaticum fʒii.

Aqua. Dest. f℥i.

M. Sum. gtts. X in water after each meal.

June 25. Has had no return of chill. Appetite good, size of spleen very much reduced, strength gaining, no indications of disease. The patient was discharged, and from this time we lost sight of him.

It will be observed that in treating this case no Quinine, Opium, Arsenic, or Strychnia was used. The Chloride of Sodium arrested the paroxysms as promptly as Quinine in ordinary doses, and the system speedily recovered its strength and vigor under the use of that agent, combined with Carb. of Iron and mineral acid. From ℥ii to ℥iii may be given to an adult without producing any unpleasant effects.

*Case Second.*—Mrs. R., an English woman, aged 30, was admitted to the Hospital June 18th. Her constitution has been robust, and her previous health good, until about three weeks since, when she become unwell, complaining at first of langor and depression, with slight chills and fever, followed in the course of a few days by diarrhœa, and still later by vomiting. At the time of her admission into the Hospital the tongue was dry, and red on the edges, with a dark crust along the centre; the teeth covered with sordes; intense thirst; some vomiting and diarrhœa, with some tenderness over the epigastric, and the right and left hypochondriac regions; the pulse small and weak; some pain in the head; a disinclination to motion of any kind, and extreme nervous prostration.

In the absence of Prof. Herrick, Prof. Evans prescribed for the case. He remarked that the case was of a Typhoid character, requiring careful and judicious nursing, with the palliation of aggravating symptoms, rather than active and energetic treatment.

The most urgent symptom of the case before us, is vomiting, which is attended with much acidity of the stomach. To correct this ℥i of Bi. Carb. Soda was ordered to be dissolved in f℥i of water, a table spoonful to be given every fifteen or twenty minutes until the acidity was corrected or the vomiting ceased. In addition, the patient was ordered to take mutton broth, strongly seasoned with Chloride of Sodium.

June 19. General appearance somewhat improved; tongue not as dry; vomiting ceased very soon after commencing the use of the Bi. Carb. of Soda; slept well during the night. Prof. H. prescribed Chloride of Sodium gr. x three times daily, Quinine gr. v at night, with the mutton broth as yesterday.

June 20. Still improving; continue treatment.

June 21. The bowels have not moved since day before yesterday; the tongue begins to be moist, and the black coat breaking up. Ordered Pil. Hydrarg. grs. v, this morning; continue the Chloride of Sodium and the Quinine.

June 23. Tenderness of the bowels entirely removed; evacuations regular and natural; appetite improving; pulse full and soft; continue the treatment.

June 26. The improvement since the last date has been constant and rapid. The patient is able to walk about the ward with ease, feels perfectly well, has a good appetite, and complains only of fatigue after exercise. She was discharged with directions to keep quiet for a few days, take a good nourishing diet, and use an abundance of *salt.*

To give in detail the Professor's views in regard to the modus operandi of Chloride of Sodium, in cases like the above, would require more space than we feel at liberty to occupy. Its use in Typhoid cases may be regarded as a modification of the Alkaline treatment. This salt is selected, not only because it prevents decomposition of the blood, but also from the fact that in that disease, as well as Pneumonia, the chlorides in general, and especially of Sodium, are deficient in the excretions. It is hoped that others may be induced to try it, marking carefully its effects upon the system, whether favorable or not, in order that its true Therapeutic character may be determined.

